# The Full Capacity of AICAR to Reduce Obesity-Induced Inflammation and Insulin Resistance Requires Myeloid SIRT1

**DOI:** 10.1371/journal.pone.0049935

**Published:** 2012-11-21

**Authors:** Zhenggang Yang, Xianfeng Wang, Yin He, Ling Qi, Liqing Yu, Bingzhong Xue, Hang Shi

**Affiliations:** 1 Department of Internal Medicine, Wake Forest University School of Medicine, Winston-Salem, North Carolina, United States of America; 2 State Key Laboratory of Infectious Disease Diagnosis and Treatment, First Affiliated Hospital of Zhejiang University, Hang Zhou, China; 3 Graduate Program in Genetics and Development, Cornell University, Ithaca, New York, United States of America; 4 Division of Nutritional Sciences, Cornell University, Ithaca, New York, United States of America; 5 Department of Animal Sciences, University of Maryland, College Park, Maryland, United States of America; 6 Department of Biology, Georgia State University, Atlanta, Georgia, United States of America; University of Padova, Italy

## Abstract

Chronic Inflammation is a key link between obesity and insulin resistance. We previously showed that two nutrient sensors AMP-activated protein kinase (AMPK) and SIRT1 interact to regulate macrophage inflammation. AMPK is also a molecular target of 5-aminoimidazole-4-carboxamide-1-β-D-ribofuranoside (AICAR), which has been shown to reduce insulin resistance in various animal models. Here we aim to determine whether the therapeutic effects of AICAR against insulin resistance involve its anti-inflammatory function, which requires macrophage SIRT1. Long-term administration of low-dose AICAR significantly suppressed adipose inflammation in established diet-induced obese mice. This was associated with improved glucose homeostasis and insulin sensitivity without changes of body weight. In contrast, SIRT1 deletion in myeloid SIRT1 knockout (MSKO) mice increased infiltration of classically activated M1 macrophages and decreased alternatively activated M2 macrophages in adipose tissue. As a result, MSKO mice on high fat (HF) diets exhibited impaired insulin signaling in skeletal muscle, fat, and liver, and developed systemic insulin resistance in glucose tolerance tests, insulin tolerance tests, and hyperinsulinemic-euglycemic clamp experiments. Interestingly, the beneficial effects of AICAR on adipose inflammation and insulin sensitivity were absent in MSKO mice fed HF diets, suggesting that the full capacity of AICAR to antagonize obesity-induced inflammation and insulin resistance requires myeloid SIRT1. In summary, AICAR negatively regulates HF diet-induced inflammation, which requires myeloid SIRT1, thereby contributing to the protection against insulin resistance. Myeloid SIRT1 is a therapeutic target of the anti-inflammatory and insulin-sensitizing effects of AICAR.

## Introduction

Chronic Inflammation is a key link between obesity and insulin resistance/type 2 diabetes [1]. Adipose tissue plays a key role in the generation of inflammatory responses and mediators in obesity [1], [2]. Recent studies have shown that obese adipose tissue exhibits increased infiltration of macrophages, and moreover, that macrophages may be a significant source of the inflammation [3], [4]. Many genetic studies support the notion that macrophage inflammation is a key component of obesity-induced inflammation and insulin resistance [5], [6], [7], [8]. However, the fundamental mechanisms responsible for the altered inflammatory programs in obesity remain elusive.

Excess nutrients (lipids and glucose), circulating levels of which are commonly elevated in obesity due to dietary sources or expanded adipose tissue, are highly detrimental in the pathological development of obesity-associated metabolic disorders, including insulin resistance and type 2 diabetes [9], [10]. Potential mechanisms whereby nutrient overexposure causes insulin resistance have been explored, and nutrient-induced metabolic stress and inflammation has emerged as a plausible mechanism [9], [10]. We reason that the excess nutrients can be sensed by the nutrient sensor AMP-activated protein kinase (AMPK), which may function as a cellular link between nutrient metabolism and inflammation [11]. Indeed, we have shown that the expression, activity and signaling of the major isoform α1AMPK in adipose tissue and macrophages are substantially down-regulated by inflammatory stimuli and in nutrient-rich conditions, and that AMPK negatively regulates lipid-induced inflammation in macrophages [11]. The role of AMPK in regulation of obesity-induced inflammation and insulin resistance were first reported in an elegant genetic study by Galic et al [12]. They convincingly demonstrated that disruption of AMPK signaling in hematopoietic derived cells including macrophages via bone marrow transplantation increased adipose macrophage inflammation and hepatic insulin resistance [12]. AMPK is also a molecular target of 5-aminoimidazole-4-carboxamide-1-β-D-ribofuranoside (AICAR), which has been shown to reduce insulin resistance in various animal models [13], [14], [15]. Our observations raise an interesting question as to whether the therapeutic effects of the AMPK agonist AICAR to reduce insulin resistance in various animal models [13], [14], [15] may depend on reduction of obesity-induced inflammation.

Like AMPK, SIRT1 also regulates inflammatory signaling in various cells [11], [16], [17]. Recent studies directly linked SIRT1’s anti-inflammatory effects in adipocytes and macrophages to improved insulin sensitivity [18], [19], [20]. We have recently shown that α1AMPK activation mimics the effect of SIRT1 on deacetylation of NF-κB, and the full capacity of AMPK to deacetylate NF-κB and inhibit its signaling requires SIRT1 [11]. These observations raise another interesting question as to whether macrophage SIRT1 is required for the protective effects of AICAR against obesity-induced inflammation and insulin resistance.

In the present study, we intend to investigate whether the therapeutic effects of AICAR against insulin resistance involve its anti-inflammatory function, which requires macrophage SIRT1. To address this question, we examined the effects of long-term AICAR administration on adipose inflammation as well as insulin sensitivity in established DIO mice. We also generated the myeloid SIRT1 knockout (MSKO) mice, and examined novel inflammatory pathways such as macrophage alternative polarization and endoplasmic reticulum (ER) stress, two pathways underlying macrophage inflammation that can not be solely explained by altered NF-κB signaling in SIRT1-deficient macrophages. We further thoroughly characterized tissue-specific and systemic insulin sensitivity of MSKO mice using comprehensive approaches such as *in vivo* insulin signaling and hyperinsulinemic-euglycemic clamps. Finally, we performed a long-term AICAR administration to MSKO mice and characterized the phenotypes of adipose macrophage inflammation and insulin sensitivity in these mice to address the main hypothesis that myeloid SIRT1 is a therapeutic target of the anti-inflammatory and insulin-sensitizing effects of AICAR.

## Results

### Long-term AICAR Administration Decreases Adipose Inflammation and Insulin Resistance in Established DIO Mice

**Figure 1:**
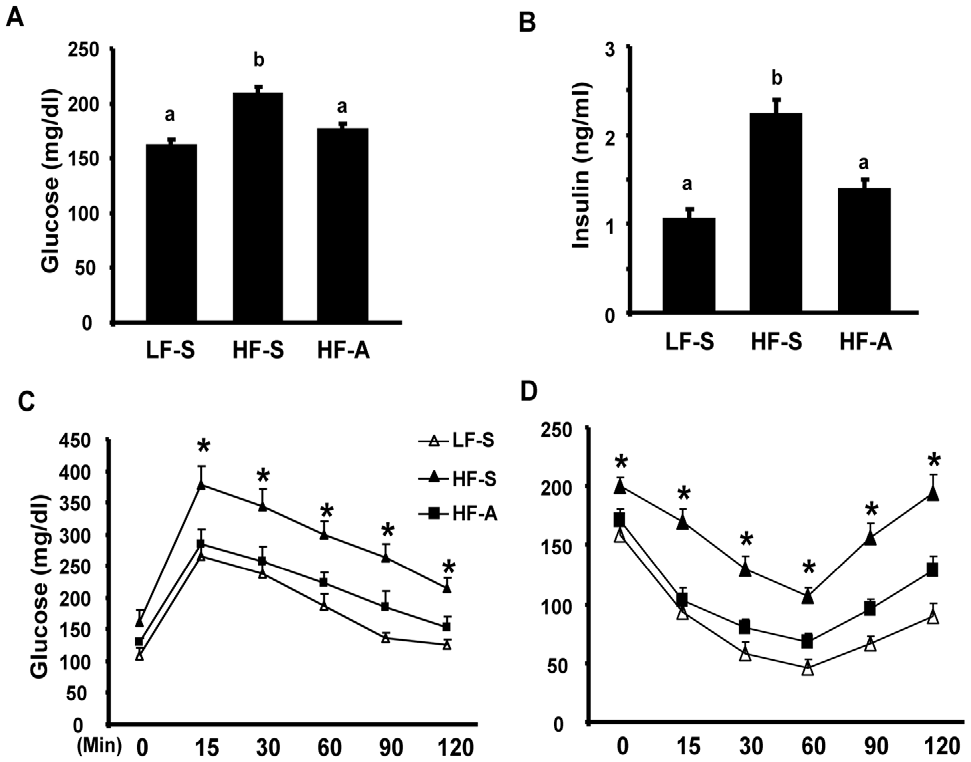
AICAR treatment ameliorates insulin resistance in diet-induced obese (DIO) mice. 6-week-old male C57BL/6J (B6) mice were placed on either low fat (LF) or high fat (HF) diets for 24 weeks to establish a control or a DIO model. Established DIO mice were randomly assigned to receive saline or AICAR (150 mg/kg) injection i.p. daily for five weeks. (A–B), Fed glucose (A) and insulin (B) levels were measured after 5 weeks of treatment. Data are expressed as mean±SE, n = 8. Groups labeled with different superscripts are statistically different from each other, p<0.05. (C–D), GTT (C) and ITT (D) were performed as described in Materials and Methods. *p<0.05 vs. HF-A. LF-S: LF-diet group with saline; HF-S: HF-diet group with saline; HF-A: HF diet group with AICAR.

We first determined whether activation of AMPK pathway by the AMPK agonist AICAR protects against obesity-induced inflammation and insulin resistance. To avoid the confounding effect of AICAR on body weight and adiposity, we chose to use a lower dose of AICAR, 150 mg/kg/day. We initially tested this low dose AICAR injection on lean mice fed a low fat chow diet to determine the potential effects on body weight. We found that administration of AICAR at this dose for 5 weeks did not change body weight and epididymal fat mass (Fig. S1 A and B). The low dose of AICAR also did not change blood glucose and insulin levels and did not alter glucose tolerance and insulin sensitivity in lean mice (Fig. S1 C–F). We then administered the same lose dose of AICAR to established DIO mice that had been fed a HF diet for 24 weeks and exhibited insulin resistance. Male C57/BL6J mice were put on LF or HF diet for 24 weeks starting at 6 weeks of age. Mice on the HF diet received either saline or the low-dose 150 mg/kg AICAR i.p. daily for 5 weeks, whereas mice on the chow diet received only saline, because we have shown that low dose of AICAR had no effects on glucose homeostasis and insulin sensitivity in lean mice (Fig. S1). As expected, AICAR treatment did not change body weight and fat pad mass in HF-fed mice over 5 weeks (Fig. S2). We previously showed that α1AMPK is abundantly expressed in fat tissue, while the expression of α2AMPK is low in fat tissue [11]. We therefore measured α1AMPK activity in fat of DIO mice treated with AICAR. Consistent with our previous findings where the AMPK signaling pathway was down-regulated by HF diet, we found here that α1AMPK activity was also decreased in epididymal fat of DIO mice compared to that of LF chow diet fed mice (Fig. S3). However, AICAR injection for 5 weeks markedly stimulated α1AMPK activity in epididymal fat of DIO mice, comparable to that of LF fed animals (Fig.S3). More importantly, AICAR treatment normalized the hyperglycemia (Fig. 1A) and hyperinsulinemia (Fig. 1B) in HF-fed mice. AICAR injection also significantly improved glucose tolerance and insulin sensitivity as assessed by GTT and ITT, respectively (Fig. 1C and 1D). Our data confirm previous observations that AICAR ameliorates insulin resistance in obese animal models [13], [14], [15]. However, our low dose of AICAR treatment improved insulin sensitivity without changes of body weight and adiposity, suggesting that other beneficial effects independent of adiposity regulation may play a role in AICAR’s insulin-sensitizing effects.

**Figure 2:**
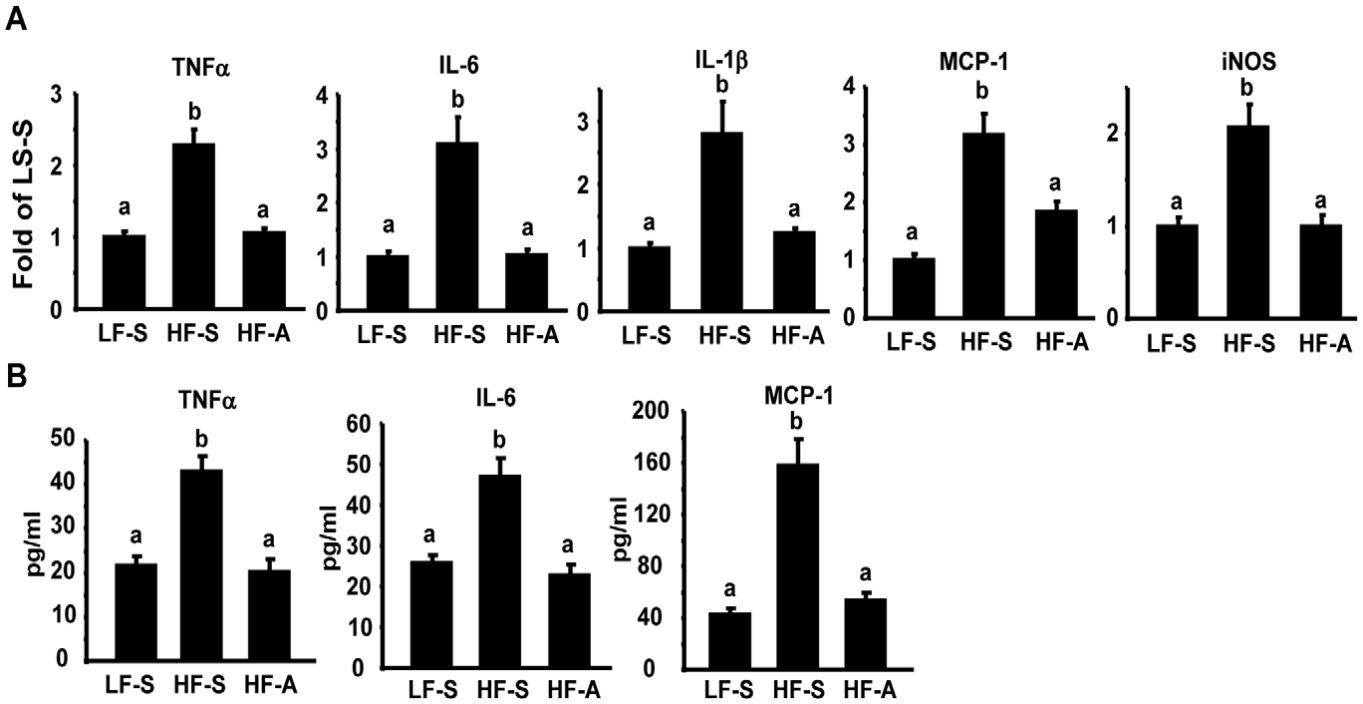
AICAR treatment suppresses inflammation in DIO mice. (A) Adipose cytokine expression in DIO mice treated with AICAR. (B) Plasma cytokine contents in DIO mice treated with AICAR. The expression of pro-inflammatory genes in adipose tissue was measured by real-time RT-PCR. Plasma cytokine contents were measured by ELISA. Data are expressed as mean±SE, n = 8. Groups labeled with different superscripts are statistically different from each other, p<0.05. LF-S: LF-diet group with saline; HF-S: HF-diet group with saline; HF-A: HF-diet group with AICAR.

Since we previously found that AMPK activation directly down-regulates macrophage inflammation [11], a major source of adipose inflammation [3], [4], we explored whether AICAR’s beneficial effects on insulin sensitivity were associated with suppressions of obesity-induced inflammation. We found that AICAR treatment in DIO mice significantly suppressed the elevated expression of pro-inflammatory genes including tumor necrosis factor α (TNFα), interleukin 6 (IL-6), IL-1β, monocyte chemoattractant protein-1 (MCP-1) and inducible nitric oxide synthase (iNOS) in epididymal fat (Fig. 2A). Similar inhibition was observed in plasma cytokine levels (TNFα, IL-6, and MCP-1) of DIO mice treated with AICAR (Fig. 2B). These data suggest that AICAR activation of AMPK suppresses adipose inflammation, which likely contributes to improved insulin sensitivity without changes of adiposity.

### Myeloid Deletion of SIRT1 Activates Inflammatory Networks via Alternative Activation

We have previously defined macrophage SIRT1 as a downstream signal in mediating AMPK’s anti-inflammatory function. To mechanistically determine the relative contribution of AICAR’s anti-inflammatory function to its insulin sensitizing effect and the role of macrophage SIRT1 in AMPK/SIRT1 axis of macrophage inflammation, we generated myeloid SIRT1 knockout (MSKO) mice by crossing SIRT1fl/fl mice [21] with lys-Cre mice (Fig. S4). There was no difference in α1AMPK activity in control and SIRT1-deficient macrophages (Fig. S5). Consistent with previous findings [20], SIRT1 deletion in macrophages promoted the expression of proinflammatory cytokines and activated the inflammatory signaling pathways such as JNK and IκB kinase (iKK)/NF-κB (Fig. S6).

**Figure 3:**
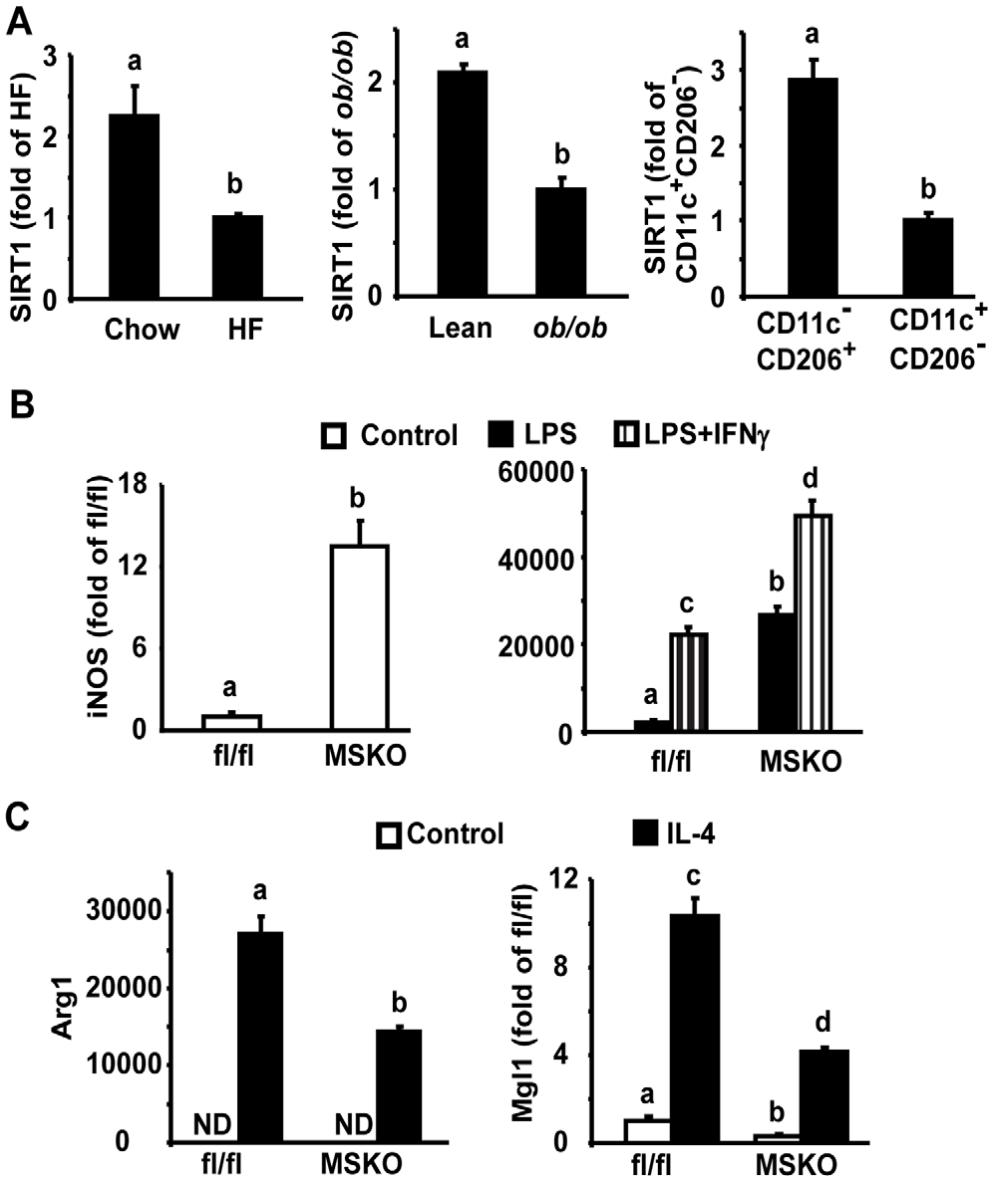
Endogenous SIRT1 regulates macrophage polarization *in vitro*. (A), SIRT1 expression is decreased in ATMs isolated from DIO (left panel) and *ob/ob* (middle panel) mice compared to their respective controls, and is higher in the M2 (F4/80^+^CD11c^−^CD206^+^) ATMs than the M1 (F4/80^+^CD11c^+^CD206^−^) population (right panel). The isolation of total ATMs and M1/M2 ATM subpopulations were described in Materials and Methods. SIRT1 mRNA was measured by real-time RT-PCR. (B–C), SIRT1 deficiency promotes M1 macrophage polarization (B), while inhibits M2 alternative activation (C) in BMDMs. BMDMs from MSKO or fl/fl mice were treated with IFN-γ (100 u/ml) plus LPS (5 ng/ml) for 24 hours in (B) or treated with IL-4 (10 ng/ml) for 48 hours in (C). The expression of M1 or M2 markers was measured by real-time RT-PCR. Data are expressed as mean±SE, n = 4–6. Groups labeled with different superscripts are statistically different from each other, p<0.05. ND: not detectable.

Adipose tissue macrophages (ATMs) play an important role in obesity-induced inflammation and insulin resistance [1]. Obesity is associated with ATM phenotypic switch from predominantly alternatively activated M2 macrophages in lean individuals to classically activated M1 macrophages in obese subjects [1]. The phenotypic changes of ATM inflammatory profile in obese animals likely involve the whole inflammatory network, whose alterations in signal transduction also occur in SIRT1-deficient macrophages. We therefore explored the physiological relevance of SIRT1 in regulation of ATM phenotypes. We first isolated ATMs from diet-induced obese (DIO) mice and genetically obese model *ob/ob* mice using MACS cell separation system with anti-F4/80 antibody and measured SIRT1 mRNA in these cells. We found that SIRT1 mRNA levels were decreased in ATMs isolated from DIO or *ob/ob* mice, compared to that of their respective controls (Fig. 3A, left and middle panels). ATMs can be distinguished into M1 and M2 macrophages by their differential expression of surface markers F4/80, CD11c and CD206/MRC1 [22], [23], [24], [25]. We isolated these M1 (F4/80^+^CD11c^+^CD206^−^) and M2 (F4/80^+^CD11c^−^CD206^+^) ATM populations using FACS with F4/80, CD11c and CD206 antibodies [22], [23], [24], [25]. We found that the expression profile of M1/M2 markers was distinct between these two populations, with high expression of the pro-inflammatory M1 markers (CD11c and iNOS) in F4/80^+^CD11c^+^CD206^−^ M1 ATMs and high expression of M2 markers (arginase 1(ARG1) and mannose receptor C type 1/CD206 (MRC1/CD206)) in F4/80^+^CD11c^−^CD206^+^ M2 ATMs (data not shown). More importantly, SIRT1 expression was much higher in F4/80^+^CD11c^−/^CD206^+^ M2 ATMs than that in F4/80^+^CD11c^+^/CD206^−^ M1 ATMs (Fig. 3A, right panel), suggesting a role for SIRT1 in the regulation of macrophage polarization.

We then determined the potential of SIRT1-deficient macrophages to become pro-inflammatory M1 macrophage [26], [27]. Bone marrow derived macrophages (BMDMs) from MSKO or fl/fl control mice were treated with the Th1 cytokine IFN-γ and the microbial trigger LPS, known inducers of M1 activation [26], and the expression of iNOS, the prototypic marker of M1 macrophages, was then measured [28]. We found that SIRT1-deficient macrophages displayed a significant increase in basal and IFN-γ/LPS-stimulated iNOS expression, suggesting that SIRT1 deletion promotes activation of M1 macrophages (Fig. 3B). On the other hand, M2 macrophages are normally induced by the Th2 cytokines such as IL-4, which typically stimulate the expression of M2 macrophage markers such as ARG1 and macrophage galactose-type c-type lectin 1 (MGL1). Our data showed that SIRT1-deficient BMDMs exhibited a significant decrease in IL-4-stimulated expression of M2 macrophage markers ARG1 and MGL1 (Fig. 3C), suggesting that SIRT1 deficiency inhibits alternative activation of M2 macrophages. In sum, our data demonstrate that macrophage SIRT1 regulates macrophage polarization by exerting a coordinated control over inhibition of M1 and stimulation of M2 macrophage activation.

**Figure 4:**
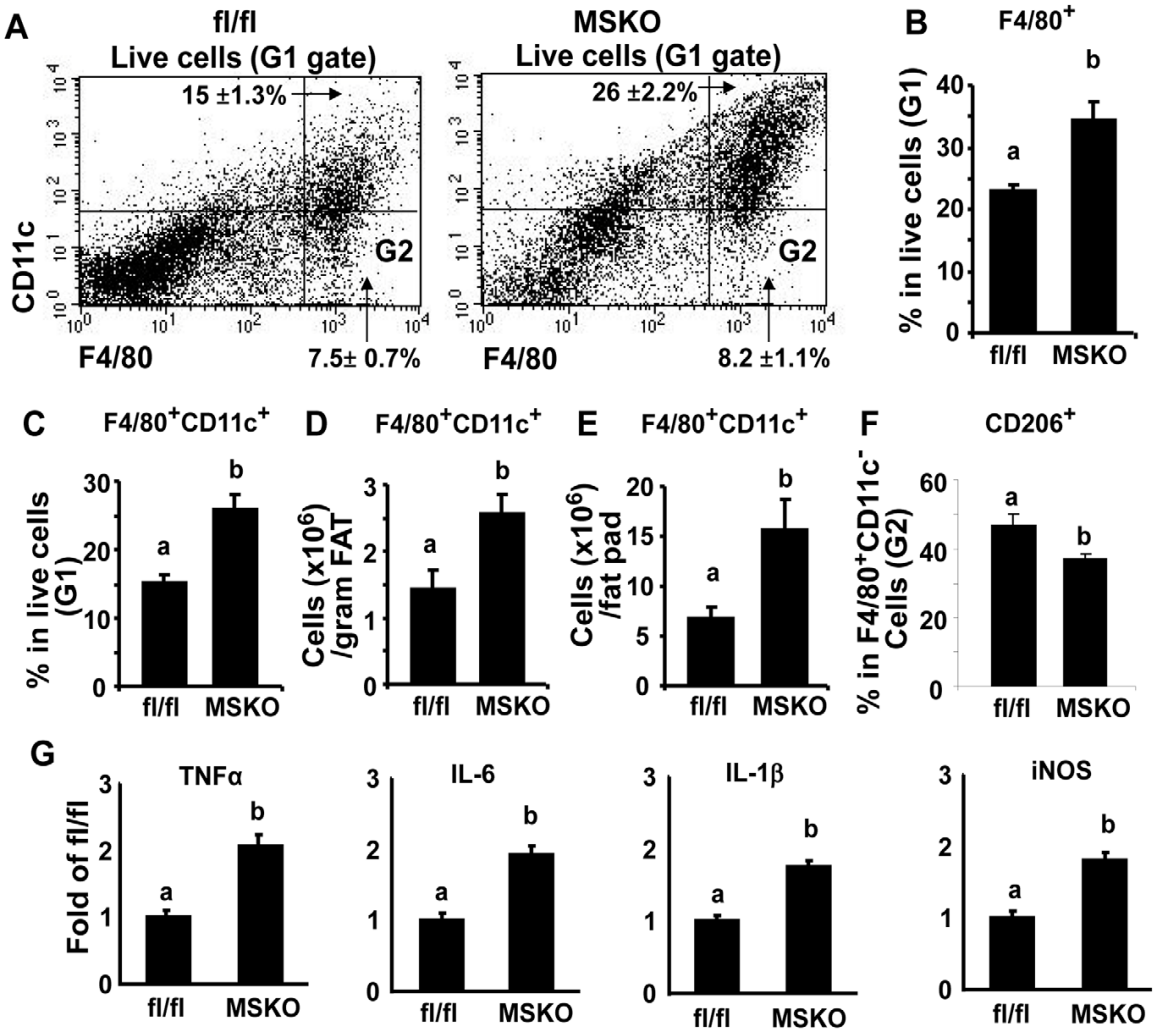
MSKO mice exhibit enhanced adipose inflammation. (A) F4/80^+^CD11c^+^ subpopulations in ATMs were increased in epididymal fat pads of MSKO mice. Isolation of adipose tissue SVF and FACS staining were described in Materials and Methods. G1: live cell gate as determined by forward scatter and side scatter; G2: F4/80^+^CD11c^−^ gate. (B–E) Quantitation of F4/80^+^ as well as F4/80^+^CD11c^+^ ATM subpopulations in epididymal fat pads. Data were presented as percentage of cells in live cells (B–C), number of cells per gram of fat (D) and number of cells per fat pad (E). (F) Percentage of CD206^+^ M2 macrophages within F4/80^+^CD11c^−^ population (G2) is reduced in epididymal fat pad of MSKO mice compared to that of fl/fl mice. (G) The expression of pro-inflammatory genes is increased in epididymal fat of MSKO mice. The expression of target genes was measured by real-time RT-PCR. Data are expressed as mean±SE, n = 5–8. Groups labeled with different superscripts are statistically different from each other, p<0.05.

To further explore the role of myeloid SIRT1 in regulation of ATM phenotypic switch in obesity-induced inflammation, we put MSKO and their fl/fl littermates on HF diets. There was no significant difference in body weight or epididymal fat mass between fl/fl control and MSKO mice on HF diets (data not shown). Using *FACS* analysis, we found that the percentage of F4/80^+^ macrophages were significantly increased in SVF cells isolated from epididymal fat pad of MSKO mice compared to fl/fl mice (Fig. 4A and 4B), indicating increased macrophage infiltration into adipose tissue. We have shown above that SIRT1-deficient macrophages have a very strong tendency to become classically activated M1 macrophages. Consistent with this, we found that the number of M1 macrophages (F4/80^+^CD11c^+^ cells) in epididymal fat of MSKO mice were also higher than that of fl/fl control mice, as indicated by the percentage of F4/80^+^CD11c^+^ cells in SVF (Fig. 4A and 4C), per gram of fat (Fig. 4D) and per epididymal fat pad (Fig. 4E). In contrast, percentage of CD206^+^ M2 macrophages within F4/80^+^CD11c^−^ population was significantly decreased in epididymal fat pad of MSKO mice compared to that of fl/fl control mice (Fig. 4F). Further experiment showed that the expression of pro-inflammatory genes such as TNFα, IL-6, IL-1β and iNOS was dramatically increased in epididymal fat from MSKO mice compared to control mice (Fig. 4G). These data suggest that myeloid SIRT1 deficiency regulates macrophage polarization by a coordinated control over promotion of M1 macrophage conversion and inhibition of M2 macrophage activation, which results in increased adipose tissue inflammation in obesity. As a result, MSKO mice on high fat (HF) diets exhibited impaired insulin signaling in skeletal muscle, fat, and liver (Fig. S7), and developed systemic insulin resistance in glucose tolerance tests, insulin tolerance tests, and hyperinsulinemic-euglycemic clamp experiments (Fig. S8).

### Myeloid SIRT1 Deficiency Prevents the Full Ability of AICAR to Reduce Inflammation and Insulin Resistance

**Figure 5:**
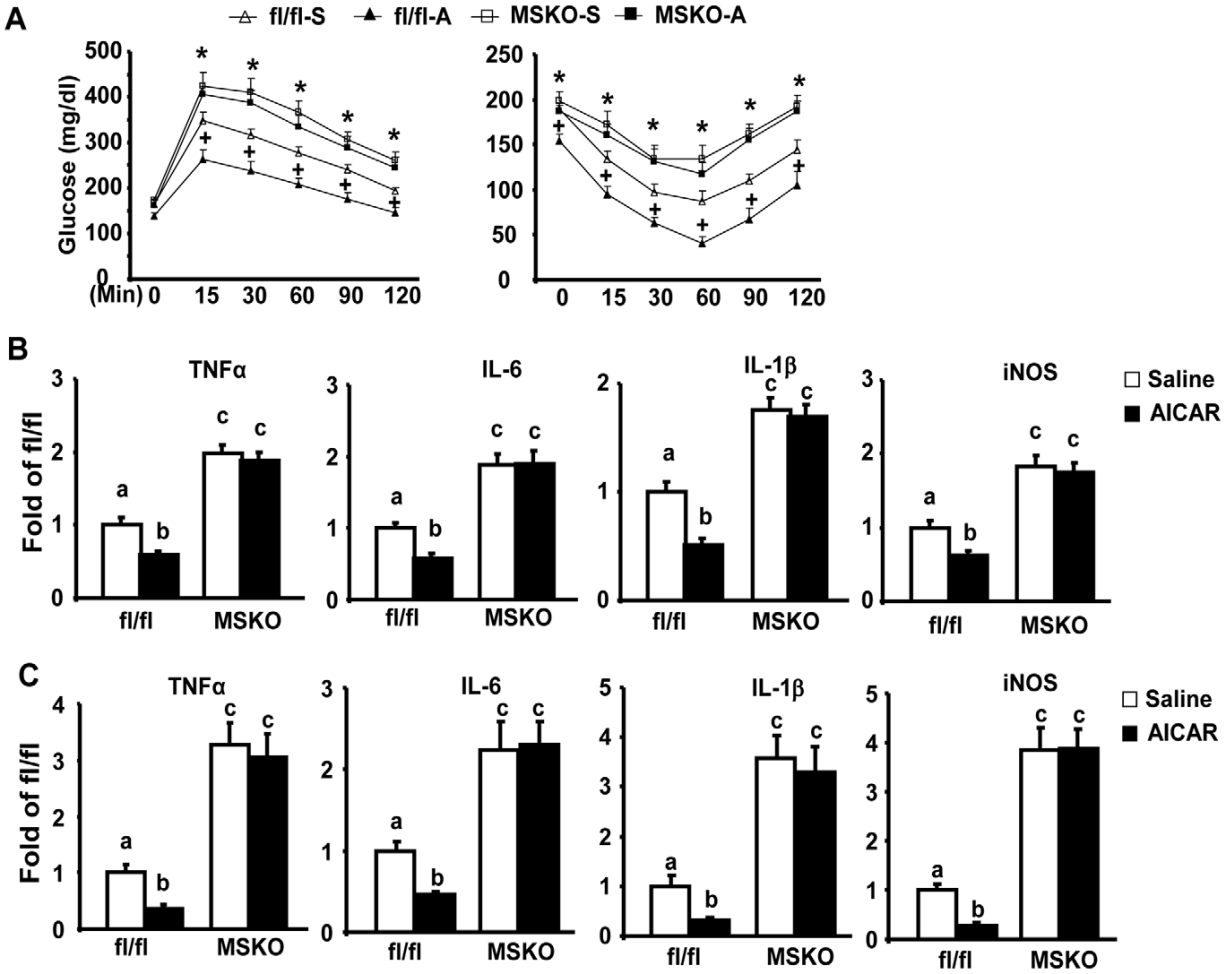
AICAR reduces insulin resistance and adipose inflammation in control fl/fl mice, but not in MSKO mice. (A) AICAR treatment ameliorates HF diet-induced insulin resistance in control fl/fl mice but not in MSKO mice. GTT (left panel) and ITT (right panel) were performed as described in Materials and Methods. +p<0.05 for fl/fl-A vs. fl/fl-S; *p<0.05 for MSKO-S and MSKO-A vs. fl/fl-S. (B–C), AICAR treatment reduces the expression of pro-inflammatory genes in epididymal fat (B) and isolated ATMs (B) in control fl/fl mice, but not in MSKO mice. ATMs from MSKO or control fl/fl mice were isolated as described in Materials and Methods. The expression of pro-inflammatory genes was measured by real-time RT-PCR. Data are expressed as mean±SE, n = 6–8. Groups labeled with different superscripts are statistically different from each other, p<0.05.

To investigate whether the therapeutic effects of AICAR against insulin resistance involve its anti-inflammatory function and work through macrophage SIRT1, we administrated AICAR to both MSKO and fl/fl control mice fed HF diets. AICAR injection significantly improved glucose tolerance and insulin sensitivity assessed by GTT and ITT in fl/fl control mice, while AICAR was not as effective in MSKO mice (Fig. 5A). Similarly, AICAR treatment decreased pro-inflammatory gene expression in both epididymal adipose tissue and isolated ATMs in control mice, but not in MSKO mice (Fig. 5B and 5C).

## Discussion

This study was designed to test the hypothesis that the therapeutic effects of the AMPK agonist AICAR against insulin resistance involve its anti-inflammatory function, which requires macrophage SIRT1. The plausibility of this hypothesis was driven by several prior findings on AMPK’s anti-inflammatory functions and AICAR’s beneficial effects on insulin resistance/type 2 diabetes. First, we and others have shown that AMPK plays an important role in the regulation of macrophage inflammation [11], [12], [17]. Second, activation of AMPK by AICAR has been shown to improve insulin sensitivity and glucose homeostasis [13], [14], [15]. It is not clear, however, whether the anti-inflammatory function of AMPK contributes to its insulin-sensitizing effects. We have previously shown that AMPK’s anti-inflammatory function depends on macrophage SIRT1 [11]. We therefore generated MSKO mice, whose insulin resistant phenotype should mainly originate from inflammation due to myeloid-specific deficiency of SIRT1. We then administrated AICAR to MSKO mice to test our hypothesis. We reasoned that if quenching inflammation is required for the full strength of AICAR to prevent insulin resistance, AICAR will not be effective in prevention of insulin resistance in MSKO mice, since AICAR likely fails to suppress the enhanced inflammation due to the absence of SIRT1. Indeed, we found that AICAR injection was able to suppress inflammation and reduce insulin resistance in control mice but not in MSKO mice. It has been known for years that AICAR has insulin-sensitizing effects [13], [14], [15]. However, the pleiotropic effects of AICAR in multiple metabolic tissues have made it difficult to determine the contribution of AMPK’s anti-inflammatory capacity to its insulin-sensitizing effects. Instead, these beneficial effects are largely attributed to AMPK actions on glucose and lipid metabolism in skeletal muscle and liver [29]. Our data clearly support the hypothesis that the full capacity of AICAR to reduce insulin resistance requires its inflammation-suppressing ability as an essential component, in addition to other beneficial effects including lipid and glucose metabolism.

Although in our study we used a low dose of AICAR that did not affect the mouse adiposity, a confounding factor often seen to affect insulin sensitivity, AICAR administration still likely exerted its insulin-sensitizing effects through direct regulation of energy metabolism in muscle and liver. In addition, AICAR may regulate inflammation independent of AMPK [30]. Additional genetic studies, involving deletion of AMPK in specific tissues, are required to separate the systemic effects on energy metabolism from the direct effect of anti-inflammation, and to determine, to what extent, the contribution of AMPK’s anti-inflammation to its insulin-sensitizing functions. As such, the macrophage is a very good target tissue to address whether the anti-inflammatory effect of AMPK is required for its insulin sensitizing and glucose-reducing effects. Indeed, Steinberg’s group was the first to investigate the role of macrophage AMPK in regulation of obesity-induced inflammation and insulin resistance [12]. They showed that genetic deletion of macrophage AMPK β1 subunit in hematopoietic cells including macrophages via bone marrow transplantation enhanced adipose tissue macrophage inflammation and insulin resistance [12]. This study discovers the importance of macrophage AMPK in regulation of obesity-induced inflammation and insulin resistance.

During the course of characterizing the phenotypes of MSKO mice, Schug et al reported a similar mouse model of myeloid SIRT1 deletion [20]. Our findings agree with the majority of the conclusions of that study, including the activated inflammation in macrophages with SIRT1 deficiency. However, we have explored the other potential mechanisms underlying the enhanced inflammation in SIRT1-deficient macrophages. Schug and colleagues attribute the enhanced inflammation in SIRT1-deficient macrophages mainly to the hyperacetylation of the NF-κB subunit p65 at lysine 310 (K310) [20]. Although acetylation of p65 at lysine 310 is a logical target due to previous findings on the ability of SIRT1 to deacetylate this lysine site and suppress NF-κB transcriptional activity [16], we believe that the pro-inflammatory phenotype of SIRT1-deficient macrophages may not be solely explained by lysine 310 hyper-acetylation. In fact, the very first study targeting the role of lysine acetylation in the regulation of p65 functions revealed that acetylation of lysine 310 is required for full transcriptional activity of p65, but has no effects on DNA binding ability of p65 [31]. Both Schug’s and our ChIP assays indicate that macrophage SIRT1 deficiency increases p65 DNA binding to its consensus promoters [20], which may not be attributed to lysine 310 hyper-acetylation. Moreover, we found that SIRT1 deletion promotes iKKα/β phosphorylation, an upstream signal of p65 nuclear translocation, and also stimulates the phosphorylation of JNK, an inflammatory signal that parallels the iKK/NF-κB pathway. All these data suggest that SIRT1 deficiency alters the global inflammatory networks. We therefore explored the inflammatory pathways involving macrophage alternative activation, which has been known to regulate systemic inflammation and play important roles in the development of metabolic disorders [1], [32]. Macrophage SIRT1 may be involved in macrophage alternative activation. We found that SIRT1 expression is higher in anti-inflammatory M2 macrophages than pro-inflammatory M1 macrophages, and that SIRT1 deficiency coordinately stimulates M1 macrophage conversion and inhibits M2 macrophage alternative activation. As a result, myeloid deletion of SIRT1 increases infiltration of classically activated M1 macrophages and decreases alternatively activated M2 macrophage content in fat. On the other hand, ER stress has emerged as a key upstream signal that activates macrophage inflammatory networks, including both JNK and NF-κB [32]. We found that SIRT1 deficiency elevated the total protein and phosphorylation of IRE1α (Fig. S9), a key ER stress sensor, in response to saturated fatty acid stearate (C18) and thapsigargin (Tg) (Fig. S8A and S8B middle), two potent inducers of macrophage ER stress [33], [34]. In sum, our data demonstrate that the altered macrophage polarization and probably ER stress pathways may contribute to the pro-inflammatory phenotype featuring activated systemic inflammatory networks in SIRT1-deficeint macrophages. Although the activated NF-κB pathway (through p65 hyperacetylation at K310) itself may partially explain the M1 macrophage tendency in SIRT1-deficient macrophages, further studies will be required to address how SIRT1 regulates macrophage polarization and ER stress pathways.

AMPK and SIRT1 share striking similarities in nutrient sensing and regulation of energy metabolism. Recent studies have disclosed a crosstalk between these two in regulation of metabolic pathways. For instance, AMPK can be an upstream signal to increase SIRT1 activity via inducing fatty acid oxidation and increasing the agonist NAD^+^ levels, leading to the deacetylation and activation of PGC-1α in muscle [35]. On the other hand, SIRT1 can also be in driving position to activate AMPK via deacetylating and activating LKB1, the upstream kinase of AMPK [36], [37]. No matter which one is the upstream or downstream signal between the two, AMPK and SIRT1 are coordinately regulated and cooperate to regulate downstream pathways. It appears that AMPK and SIRT1 can activate each other and feed off ensuing signaling between them. Which one is the upstream or downstream signal may depend on different types of cells or biological pathways. In regulation of the macrophage inflammation, we previously found that AMPK antagonizes inflammation through SIRT1 by increasing the SIRT1 activator NAD^+^ [11]. Interestingly, Galic et al demonstrated a key role of fatty acid oxidation in mediating AMPK inhibition of macrophage inflammation [12]. Fatty acid oxidation appears to be a novel mechanism underlying nutrient-induced inflammation in macrophages and fits well into the scenario where AMPK interacts with SIRT1 to regulate macrophage inflammation, because increasing fatty acid oxidation may enhance cellular NAD^+^ content, which may further activate SIRT1. In the present study, we also found that myeloid SIRT1 may serve as the downstream signal that mediates the anti-inflammatory of the AMPK agonist AICAR *in vivo*. It is likely that activation of AMPK may induce fatty acid oxidation and increase cellular NAD^+^, which further lead to activation of SIRT1.

In summary, our data demonstrate that AICAR treatment decreases adipose macrophage inflammation, thereby contributing to the protection against HF diet-induced insulin resistance. In contrast, myeloid deletion of SIRT1 increases macrophage inflammation through promotion of pro-inflammatory M1 polarization coupled with inhibition of M2 activation. The beneficial effects of AICAR in antagonizing inflammation and insulin resistance require myeloid SIRT1. We conclude that myeloid SIRT1 is a therapeutic target of the anti-inflammatory and insulin-sensitizing effects of AICAR. The anti-inflammatory property of AMPK and SIRT1 may contribute to their beneficial effects in antagonizing obesity-induced insulin resistance.

## Materials and Methods

### Animals

The animal studies were approved by the institutional animal care and use committee of the Baptist Medical Center at Wake Forest University. For AICAR injection studies, 6-week-old male C57BL/6J (B6) mice (Jackson Laboratories, Bar Harbor, ME) were placed on either a low fat (LF) (Research Diets D12450B, 10% calories from fat, Research Diets Inc., New Brunswick, NJ) or a HF diet (Research Diets D12492, 60% calories from fat, Research Diets Inc.) for 24 weeks to establish diet-induced obese (DIO) mouse model. DIO mice were randomly assigned to receive either saline or AICAR (Toronto Research Chemicals, North York, Ontario) injection intraperitoneally (i.p.) daily for five weeks. At the AICAR dose of 150 mg/kg/day, we did not observe any body weight changes in established DIO mice.

Myeloid SIRT1 knockout (MSKO) mice were created by crossing SIRT1flox/flox (fl/fl) mice in which the SIRT1 allele contains a loxp-flanked exon 4 [21] (purchased from the Jackson Lab, Bar Harbor, ME, and backcrossed to C57/BL6J for 5 generations) with lysozyme Cre (lys-Cre) mice (the Jackson Lab) in which Cre is specifically expressed in myeloid lineage cells including macrophages and monocytes. MSKO mice and their fl/fl littermates were put on either a LF (Research Diets D12450B, 10% calories from fat, Research Diets Inc.) or a HF diet (Research Diets D12492, 60% calories from fat, Research Diets Inc.) for up to 28 weeks starting at 6 weeks of age. All animals were housed with a 12-h light/dark cycle in a temperature-controlled facility and had free access to water and food.

### Metabolic Measurements

Metabolic measurements were conducted as we previously described [38], [39]. Body weight was monitored weekly. Blood glucose was measured with an OneTouch Ultural Glucose meter (Lifescan, Mulpitas, CA). Serum insulin levels were measured using rat insulin enzyme-linked immunosorbent assay (ELISA) kits (Crystal Chem, Downers Grove, IL). Plasma cytokine contents were measured by ELISA kits (R&D Systems, Minneapolis, MN). Glucose tolerance test (GTT) and Insulin tolerance test (ITT) were performed as we previously described [38]. For GTT, mice were fasted overnight, and blood glucose was measured immediately before and 15, 30, 60, 90, and 120 min after an intraperitoneal (i.p.) injection of glucose (1.2–1.8 g/kg of body weight). For ITT, mice were injected intraperitoneally with 1–1.8 unit/kg of human insulin (Humulin R, Eli Lilly, Indiana, IN) after a 6-hr food removal, and glucose levels were measured at different time points (0, 15, 30, 60, 120 minutes).

### AMPK Activity

Adipose tissue lysates were immunoprecipitated with specific antibody (Upstate, Lake Placid, NY) against α1 subunit bound to protein G-Sepharose beads. The kinase activity of the immunoprecipitates was measured using “SAMS” peptide and [γ-^32^P]ATP [11].

### Insulin Signaling Studies

Mice were fasted overnight. Human insulin (10 units/kg of body weight; Humulin R) was injected intraperitonealy; 10 minutes later, mice were killed by CO_2_, and tissues were quickly collected and snap-frozen in liquid nitrogen. Tissues were stored at –80°C until processing.

### Immunoblotting and *Immunoprecipitation*

Tissues were homogenized in a modified radioimmunoprecipitation assay (RIPA) buffer. For immunoblotting, protein lysate (50–100 µg) was resolved by SDS-PAGE, and the phosphorylation and total levels of specific proteins were measured by immunoblotting. After incubating with primary antibodies, blots were incubated with Alexa Fluor 680-conjugated secondary antibodies (Invitrogen, Carlsbad, CA) and developed with a Li-COR Odyssey Infrared Imager system (Li-COR Biosciences, Lincoln, NE). Rabbit anti-IR and goat anti-β-actin polyclonal antibodies were from Santa Cruz Biotechnology Inc. (Santa Cruz, CA). Rabbit anti-IRS-1 polyclonal antibodies were from Upstate (Lake Placid, NY). Rabbit anti-phospho-IR Tyr^1162/^Tyr^1163^ polyclonal antibodies were from Invitrogen. Rabbit anti-phospho-Akt/protein kinase B (PKB) Ser^473^, anti-total Akt/PKB and anti-phospho-JNK polyclonal antibodies were from Cell Signaling Technology, Inc. (Beverly, MA). For the measurement of phosphorylation of IRS-1, tissue lysates were immunoprecipitated with IRS-1 (Upstate) antibodies, and followed by immunoblotting with phosphotyrosine 4G10 antibody (Upstate). For immunoprecipitation, 1 mg of tissue lysates was incubated overnight with appropriate antibodies and protein A agarose (Santa Cruz) at 4°C with constant gentle shaking. Agarose beads were collected by centrifugation, washed with ice-cold RIPA lysis buffer 2 times and PBS 2 times, then boiled in 2X Laemmli sample buffer for denaturation of proteins. The immunoprecipitated protein was used for immunoblotting.

The ER stress signal IRE1α phosphorylation was measured using phos-tag-based approach as previously described [40].

### RNA Extraction and Reverse Transcription (RT)-PCR

RNA was extracted using TriReagent (Molecular Research Center, Inc., Cincinnati, OH). The mRNA levels of target genes were quantified by quantitative real-time RT-PCR as described previously using Stratagene Mx3000 (Agilent Technologies, Santa Clara, CA) [38]. Primer and probe sequences for cyclophilin were: 5′-GGTGGAGAGCACCAAGACAGA-3′ (forward), 5′-GCCGGAGTCGACAATGATG-3′ (reverse), and 5′-TCCTTCAGTGGCTTGTCCCGGCT-3′ (probe). TaqMan primer/probes for all other genes were purchased from Applied Biosystems (Applied BioSystems, Foster City, CA).

### Fluorescence-activated Cell Sorting (FACS) Analysis

Isolation of adipose tissue stromal vascular cells and FACS analysis were performed as we described [38], [41]. Briefly, 1–2 g of epididymal fat were placed in Krebs-Ringer HEPES (KRH) buffer containing 10 mg/ml fatty acid–poor BSA (Sigma-Aldrich, St. Louis, MO), minced and digested in 1 mg/ml collagenase type I (Worthington Biochemical Co, lakewood, NJ) by shaking (70 Hz) at 37°C for 30 minutes. Samples were filtered through a 300 µm nylon mesh (Spectrum Laboratories, Rancho Dominguez, CA), and the resulting suspension was centrifuged at 400 *g* for 10 min to separate stromal vascular fraction (SVF) cells from adipocytes. SVF cells were incubated in erythrocyte lysis buffer (eBioScience, San Diego, CA) for 5 min and then washed with KRH-BSA buffer. Cells were then re-suspended in FACS buffer (eBioscience). A portion of the cells was counted with a hemacytometer. Based on trypan blue exclusion, the percentage of live cells per sample was usually greater than 95%. Cells were incubated in the dark on a shaker with FcBlock (eBioscience) for 30 min at 4°C and further incubated for 1 hour with allophycocyanin (APC)-conjugated F4/80, PE-conjugated CD206 (AbD Serotec, Releigh, NC), and phycoerythrin (PE)-Cy7-conjugated CD11c antibodies (eBioscience). After incubation, cells were washed with FACS buffer, fixed in 4% paraformaldehyde and analyzed with a FACS Calibur machine (BD, Franklin Lakes, NJ). FACS data were analyzed using CellQuest software (BD, Franklin Lakes, NJ).

### Isolation of Adipose Macrophages and M1 and M2 Macrophages

Isolation of adipose tissue stromal vascular cells was performed as described above. Stromal vascular cells were then incubated with rat anti-mouse F4/80 polyclonal antibodies (AbD Serotec), followed by a pull down of F4/80-positive cells with sheep anti-rat microbeads using magnetic-activated cell sorting (MACS) system according to manufacturer's instructions (Miltenyi Biotec, Auburn, CA). Isolated F4/80^+^ adipose tissue macrophages (ATMs) were used for gene expression analysis. In another experiment, stromal vascular cells were labeled with APC-F4/80, PE-Cy7-CD11c and PE-CD206 as described above, and M1/M2 macrophage subsets were isolated using BD FACSAria Cell Sorting machine (BD, Franklin Lakes, NJ).

### Hyperinsulinemic-euglycemic Clamp Study

Hyperinsulinemic-euglycemic clamp study was performed essentially as described [42]. Mice were implanted with the indwelling catheters and allowed to recover for 5 days. After an initial 5-µCi bolus, [3-^3^H]-glucose was infused at 0.05 µCi/min for 2 hrs to measure basal glucose turnover. A 2-hr hyperinsulinemic-euglycemic clamp were conducted with a prime and continuous infusion of insulin at a rate of 2.5 mU/kg/min, coupled with a variable infusion of 40% glucose to maintain blood glucose at 6 mM. Blood glucose was measured via tail bleed every 5 minutes in the 1^st^ hour to achieve stable blood glucose levels and every 10 minutes until the end of the 2-hour clamp to maintain constant blood glucose levels. The rate of whole body glucose turnover was estimated using a continuous infusion of [3-^3^H]-glucose at 0.1 µCi/min. Tissue-specific glucose uptake was estimated by a bolus administration of 10 µCi 2-deoxy-D-[1-^14^C]-glucose 45 minutes before the end of clamp experiments.

### Cell Culture

Bone marrow (BM) was flushed from the femur and tibia, dispersed, and cultured in DMEM containing 10% FBS and 30% L929 conditional medium for 8 days. Peritoneal macrophages were isolated by lavage 4 days after intraperitoneal injection of 3% thioglycollate (2 ml; Difco, BD Biosciences, San Jose, CA). The cells were plated at a density of 1.2×10^6^ cells/well in 6-well plates and cultured in RPMI 1640 medium containing 10% heat-inactived FBS. For treatment with Fatty acids to induce ER stress, stearate (Sigma-Aldrich, St. Louis, MO) was conjugated with BSA at a 4∶1 molar ratio. Stearate was first dissolved in 95% ethanol at 60°C and then was mixed with pre-warmed BSA (10%) to yield a stock concentration of 3.75 mM.

### Chromatin Immunoprecipitation (ChIP) Assay

ChIP was conducted using a ChIP assay kit (Upstate) as we previously described [11], [42]. Briefly, cells were fixed with 1% of formaldehyde and then harvested in cell lysis buffer (5 mM PIPES, 85 mM KCl, and 0.5% NP-40, supplemented with protease inhibitors, pH 8.0). The lysates were sonicated to shear genomic DNA to an average fragment length of 200–1000 bp. Lysates were centrifuged, and the supernatants were collected. The supernatants underwent overnight immunoprecipitation with anti-p65 antibody (SC-372, Santa Cruz, Santa Cruz, CA), elution, reverse cross-link, and protease K digestion. The DNAs recovered from phenol/chloroform extraction were used for PCR amplification. The TNFα promoter primer sequences are 5′-ACCCAAAGCAGCAGCCTGAG-3′ (Forward) and 5′-GGACATCCATGGGGGAGAAC-3′ (Reverse).

### Statistical Analysis

Results are presented as the mean ± S.E. Differences between groups were analyzed for statistical significance by Student’s *t*-test, analysis of variance (ANOVA) with Fischer's probable least-squares difference post hoc test or ANOVA with repeated measures as appropriate.

## Supporting Information

Figure S1 **Low dose AICAR does not affect insulin sensitivity in lean mice fed a low fat (LF) chow diet.** 6-month-old male C57BL/6J (B6) mice fed a low fat chow diet received either saline or AICAR (150 mg/kg) injection i.p. for 5 weeks. Body weight (A) was measured weekly, and epididymal fat (B) was dissected and weighted after 5-week AICAR treatment. Fed glucose (C),insulin (D), GTT (E) and ITT (F) were measured after 5 weeks of treatment. Data are expressed as mean ± SE, n = 8.
(TIF)

Figure S2 **Low dose AICAR does not change body weight and fat pad mass in established diet-induced obese (DIO) mice.** 6-week-old male C57BL/6J (B6) mice were placed on either low fat (LF) or high fat (HF) diets for 24 weeks to establish a control or a DIO model, respectively. Established DIO mice were randomly assigned to receive saline or AICAR (150 mg/kg) injection i.p. daily for five weeks. Body weight (A) was measured weekly, and epididymal fat (B) was dissected and weighted after 5-week AICAR treatment. Data are expressed as mean ± SE, n = 8. LF-S: LF diet with saline; HF-S: HF diet with saline; HF-A: HF diet with AICAR.
(TIF)

Figure S3 **AICAR stimulates α1AMPK activity in adipose tissue of DIO mice.** 6-week-old male C57BL/6J (B6) mice were placed on either low fat (LF) or high fat (HF) diets for 24 weeks to establish a control or a DIO model. Established DIO mice were randomly assigned to receive saline or AICAR (150 mg/kg) injection i.p. daily for five weeks. Epididymal fat was used for α1AMPK activity using an immune complex assay as described in the Materials and Methods. Data are expressed as mean ± SE, n = 8. Groups labeled with different superscripts are statistically different from each other, p<0.05. LF-S: LF-diet group with saline; HF-S: HF-diet group with saline; HF-A: HF diet group with AICAR.
(TIF)

Figure S4 **Generation of MSKO mice.** SIRT1 mRNA and protein expression were reduced by 80% in both peritoneal (A) and bone marrow (BM) derived macrophages (B). Peritoneal and BM derived macrophages were cultured as described in Materials and Methods. SIRT1 mRNA and protein were measured by real-time RT-PCR and western blotting, respectively. Groups labeled with different superscripts are statistically different from each other, p<0.05.
(TIF)

Figure S5 **Macrophage AMPK activity in MSKO mice.** Peritoneal macrophages were treated with AICAR (0.25 mM) for 4 hrs. α1AMPK activity was measured by an immune complex assay as described in the Materials and Methods. Data are expressed as mean ± SE, n = 4. Groups labeled with different superscripts are statistically different from each other, p<0.05.
(TIF)

Figure S6 **SIRT1 deficiency activates the inflammatory networks in macrophages.** (A) SIRT1 deficiency increases LPS-stimulated expression of pro-inflammatory genes in macrophages. Peritoneal macrophages from MSKO or fl/fl mice were treated with LPS (100 ng/ml) for 30 mins. The expression of inflammatory genes was measured by real-time RT-PCR. Data are expressed as mean ± SE, n = 4. Groups labeled with different superscripts are statistically different from each other, p<0.05. (B–D), SIRT1 deficiency increases phosphorylation of JNK (B), IKKα/β (C), and p65 DNA binding to the TNFα promoter (D). Peritoneal macrophages from MSKO or fl/fl mice were treated with LPS (100 ng/ml) for 15 or 30 mins. JNK and iKK phosphorylation were measured by western blotting and the blots were quantitated with a Li-COR Odyssey infrared Imager System (lower panels in D and C). p65 DNA binding to the TNFα promoter were measured by ChIP assays.
(TIF)

Figure S7 **MSKO mice exhibit impaired insulin signaling in muscle, fat, and liver.** Insulin signaling study was conducted in male MSKO and fl/fl mice fed HF diets for 24 weeks as described in Materials and Methods. Gastrocnemius muscle, epididymal fat and liver were collected and tyrosyl phosphorylation of IR at Tyr^1162/1163^ and serine phosphorylation of Akt at Ser-472 were measured by western blotting analysis. Tyrosyl phosphorylation of IRS1 was measured by immunoprecipitation followed by western blotting.
(TIF)

Figure S8 **MSKO mice develop insulin resistance on HF diets.** (A–B), GTT (A) and ITT (B) were performed on male MSKO and fl/fl control mice fed HF diets for 24 weeks. n = 8–12, *p<0.05 vs. fl/fl. (C–F), Glucose infusion rate (C), glucose turnover rate (D), muscle glucose uptake (E), and fat glucose uptake (F) in male MSKO and control fl/fl mice during hyperinsulinemic-euglycemic clamp study. Hyperinsulinemic-euglycemic clamp was performed as described in Materials and Methods. Data are expressed as mean ± SE, n = 5–6. Groups labeled with different superscripts are statistically different from each other, p<0.05.
(TIF)

Figure S9 **SIRT1 deficiency increases IRE1α protein and phosphorylation levels.** (A) Representative blots of IRE1α signaling. (B) Quantitation of IRE1α protein and phosphorylation. Peritoneal macrophages from MSKO or fl/fl mice were isolated by lavage 4 days after intraperitoneal injection of thioglycollate, and were treated with stearate (C: 18, 200 µM) overnight or thapsigargin (Tg) for 2 hours. IRE1α phosphorylation was measured using phos-tag-based approach as described in Materials and Methods. CON: control; BSA: bovine serum albumin. HSP90: heat shock protein 90α (internal control).
(TIF)
