# Supplementary figures and images for: The Full Capacity of AICAR to Reduce Obesity-Induced Inflammation and Insulin Resistance Requires Myeloid SIRT1
Source: PLoS One. 2012 Nov 21;7(11):e49935. doi: 10.1371/journal.pone.0049935 (PMC3503857; doi:10.1371/journal.pone.0049935)

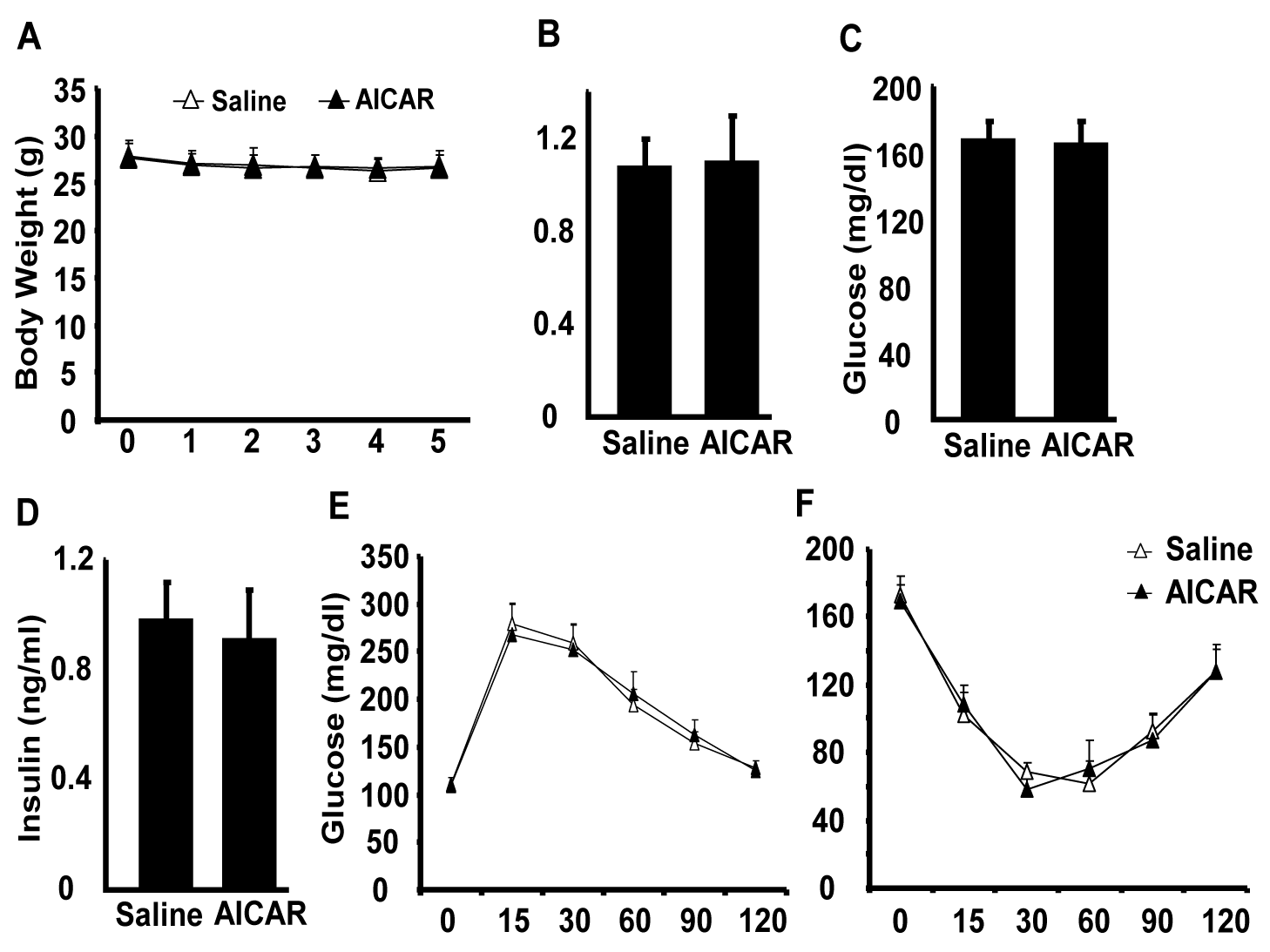

Supplement: Figure S1 — Low dose AICAR does not affect insulin sensitivity in lean mice fed a low fat (LF) chow diet. 6-month-old male C57BL/6J (B6) mice fed a low fat chow diet received either saline or AICAR (150 mg/kg) injection i.p. for 5 weeks. Body weight (A) was measured weekly, and epididymal fat (B) was dissected and weighted after 5-week AICAR treatment. Fed glucose (C),insulin (D), GTT (E) and ITT (F) were measured after 5 weeks of treatment. Data are expressed as mean ± SE, n = 8. (TIF) [file pone.0049935.s001.tif]

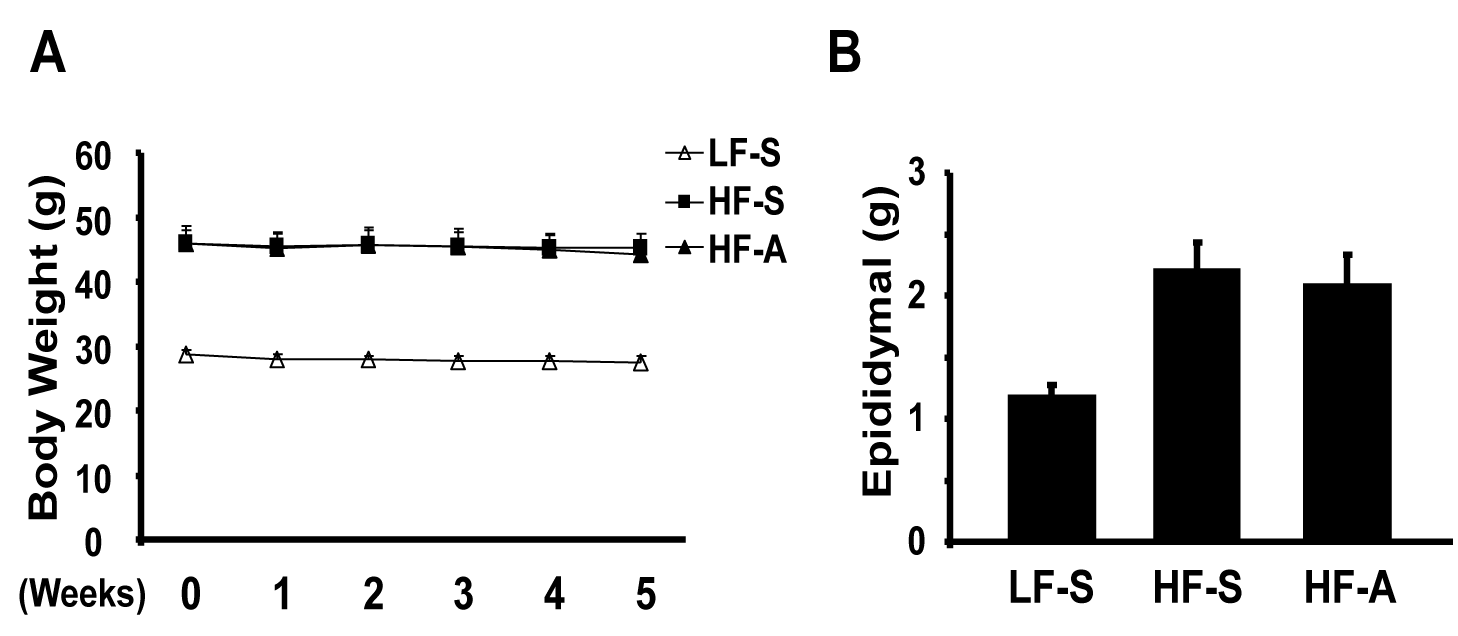

Supplement: Figure S2 — Low dose AICAR does not change body weight and fat pad mass in established diet-induced obese (DIO) mice. 6-week-old male C57BL/6J (B6) mice were placed on either low fat (LF) or high fat (HF) diets for 24 weeks to establish a control or a DIO model, respectively. Established DIO mice were randomly assigned to receive saline or AICAR (150 mg/kg) injection i.p. daily for five weeks. Body weight (A) was measured weekly, and epididymal fat (B) was dissected and weighted after 5-week AICAR treatment. Data are expressed as mean ± SE, n = 8. LF-S: LF diet with saline; HF-S: HF diet with saline; HF-A: HF diet with AICAR. (TIF) [file pone.0049935.s002.tif]

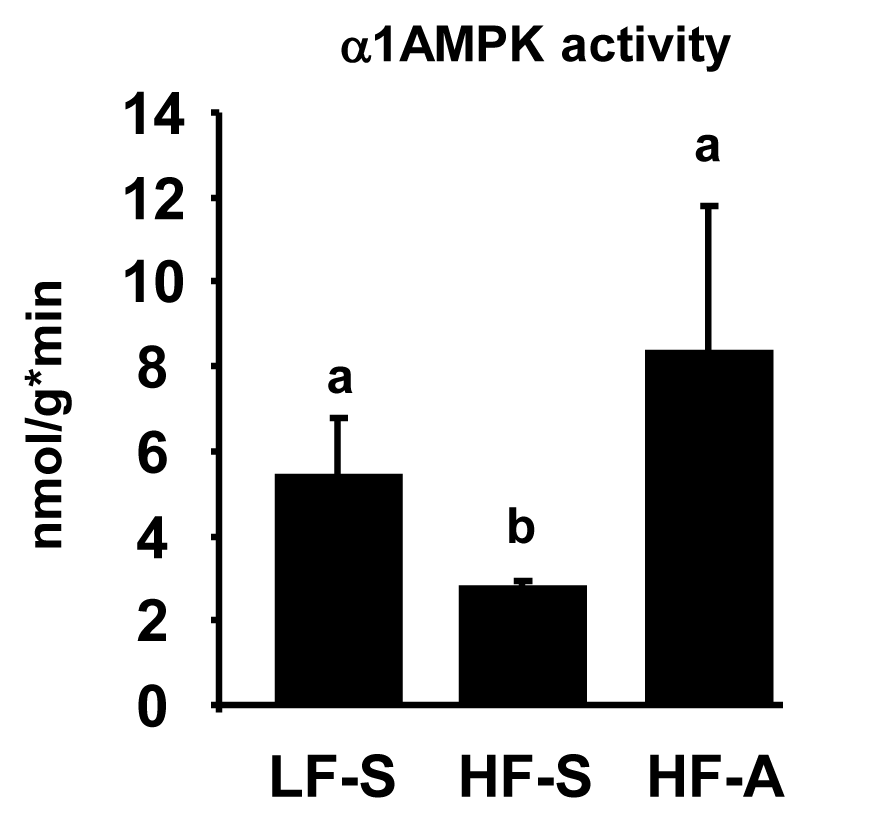

Supplement: Figure S3 — AICAR stimulates α1AMPK activity in adipose tissue of DIO mice. 6-week-old male C57BL/6J (B6) mice were placed on either low fat (LF) or high fat (HF) diets for 24 weeks to establish a control or a DIO model. Established DIO mice were randomly assigned to receive saline or AICAR (150 mg/kg) injection i.p. daily for five weeks. Epididymal fat was used for α1AMPK activity using an immune complex assay as described in the Materials and Methods. Data are expressed as mean ± SE, n = 8. Groups labeled with different superscripts are statistically different from each other, p<0.05. LF-S: LF-diet group with saline; HF-S: HF-diet group with saline; HF-A: HF diet group with AICAR. (TIF) [file pone.0049935.s003.tif]

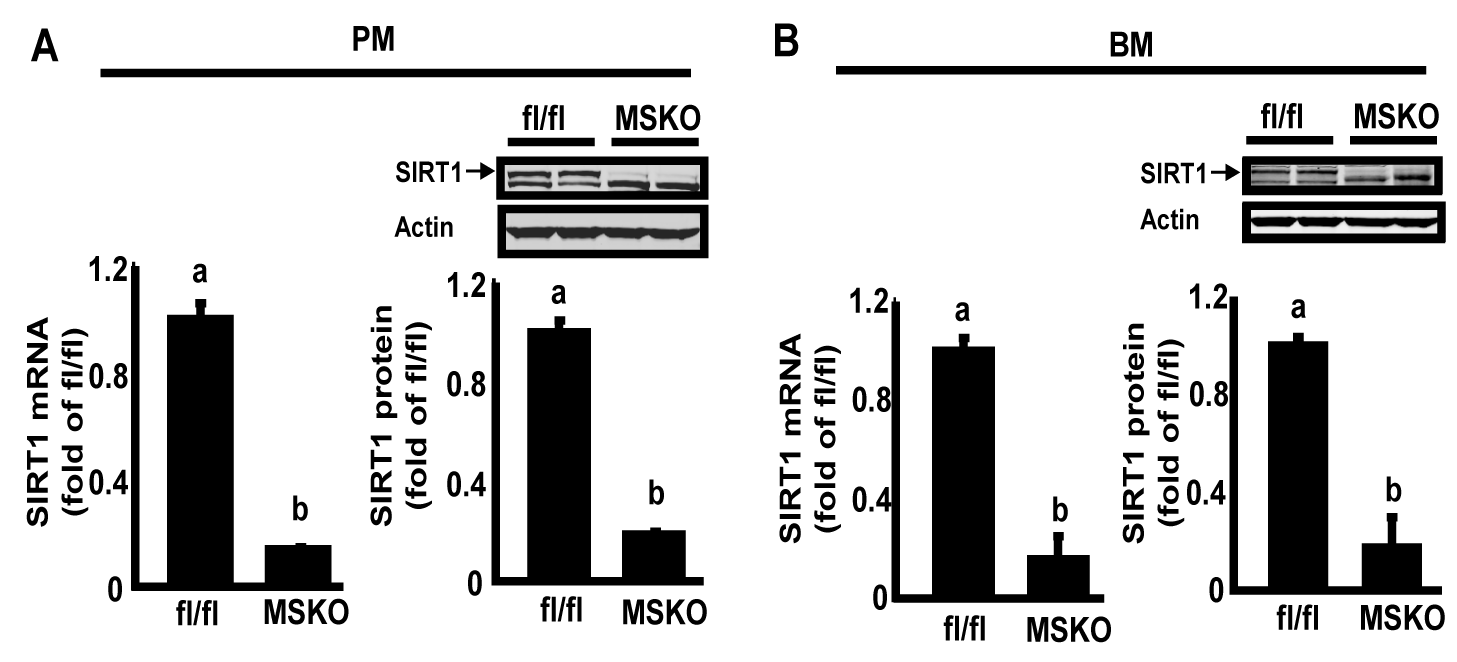

Supplement: Figure S4 — Generation of MSKO mice. SIRT1 mRNA and protein expression were reduced by 80% in both peritoneal (A) and bone marrow (BM) derived macrophages (B). Peritoneal and BM derived macrophages were cultured as described in Materials and Methods. SIRT1 mRNA and protein were measured by real-time RT-PCR and western blotting, respectively. Groups labeled with different superscripts are statistically different from each other, p<0.05. (TIF) [file pone.0049935.s004.tif]

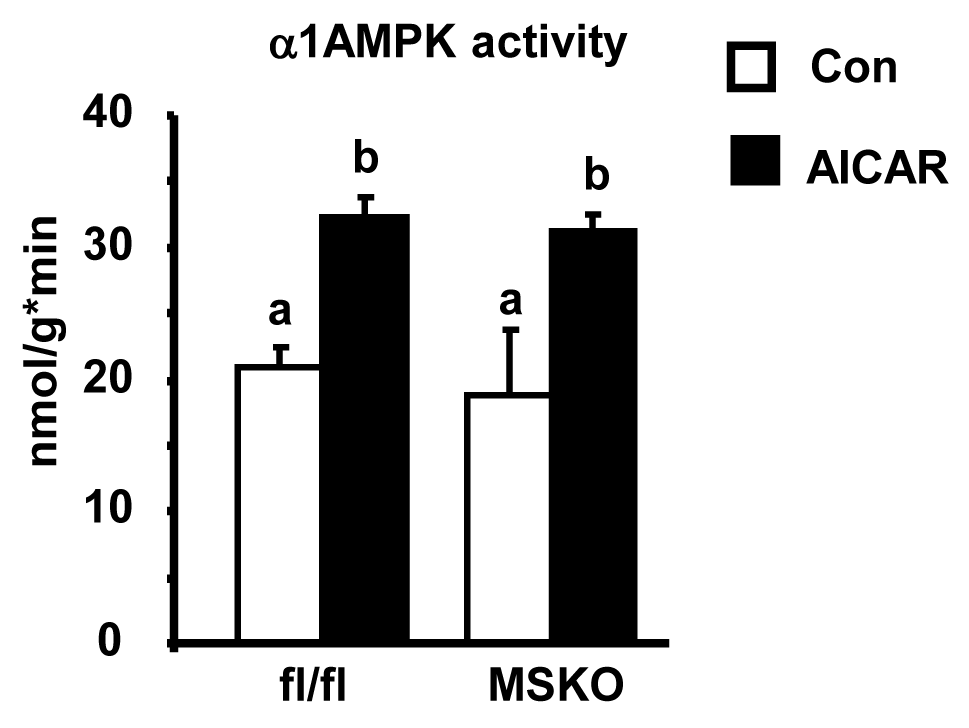

Supplement: Figure S5 — Macrophage AMPK activity in MSKO mice. Peritoneal macrophages were treated with AICAR (0.25 mM) for 4 hrs. α1AMPK activity was measured by an immune complex assay as described in the Materials and Methods. Data are expressed as mean ± SE, n = 4. Groups labeled with different superscripts are statistically different from each other, p<0.05. (TIF) [file pone.0049935.s005.tif]

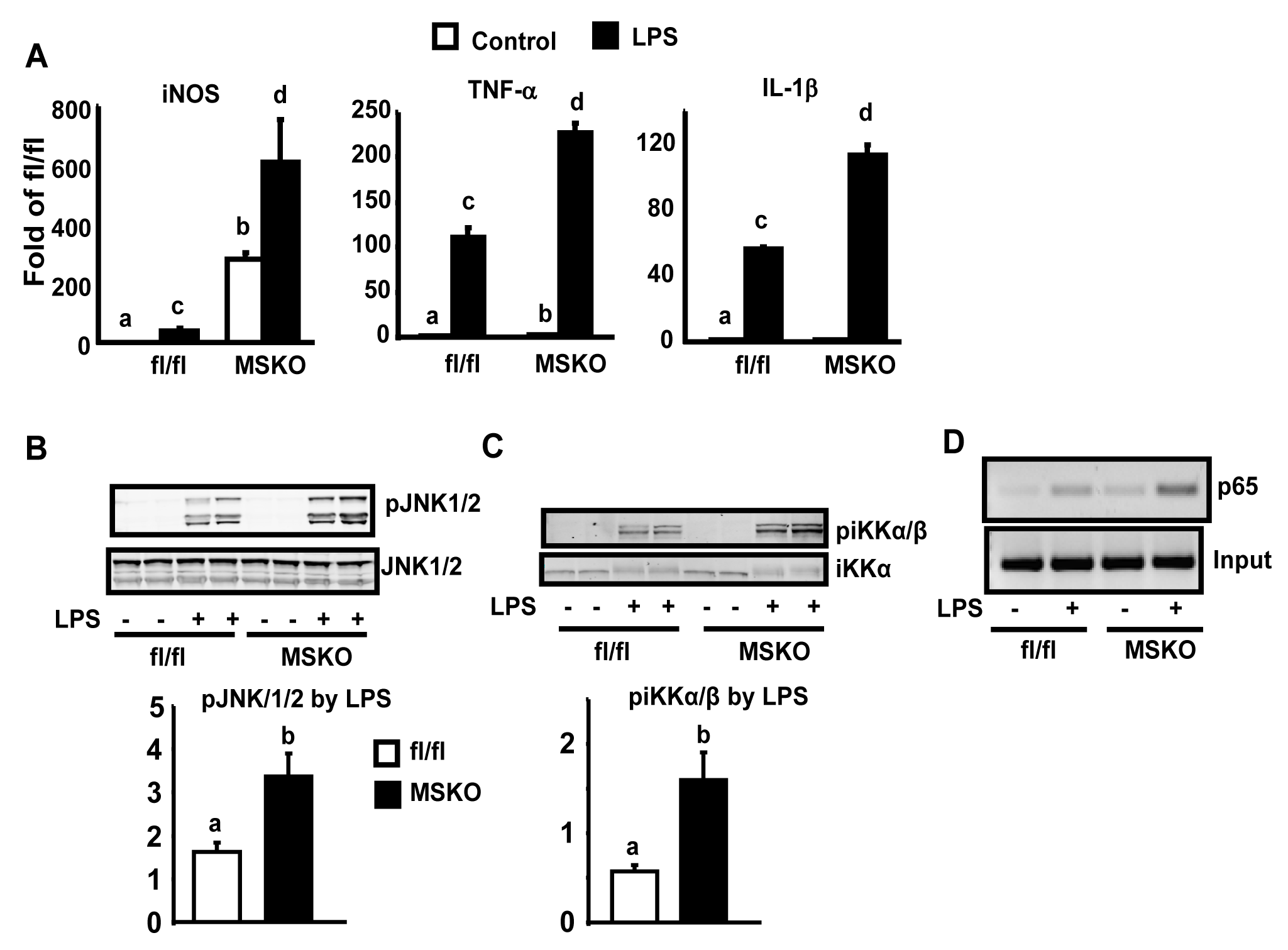

Supplement: Figure S6 — SIRT1 deficiency activates the inflammatory networks in macrophages. (A) SIRT1 deficiency increases LPS-stimulated expression of pro-inflammatory genes in macrophages. Peritoneal macrophages from MSKO or fl/fl mice were treated with LPS (100 ng/ml) for 30 mins. The expression of inflammatory genes was measured by real-time RT-PCR. Data are expressed as mean ± SE, n = 4. Groups labeled with different superscripts are statistically different from each other, p<0.05. (B–D), SIRT1 deficiency increases phosphorylation of JNK (B), IKKα/β (C), and p65 DNA binding to the TNFα promoter (D). Peritoneal macrophages from MSKO or fl/fl mice were treated with LPS (100 ng/ml) for 15 or 30 mins. JNK and iKK phosphorylation were measured by western blotting and the blots were quantitated with a Li-COR Odyssey infrared Imager System (lower panels in D and C). p65 DNA binding to the TNFα promoter were measured by ChIP assays. (TIF) [file pone.0049935.s006.tif]

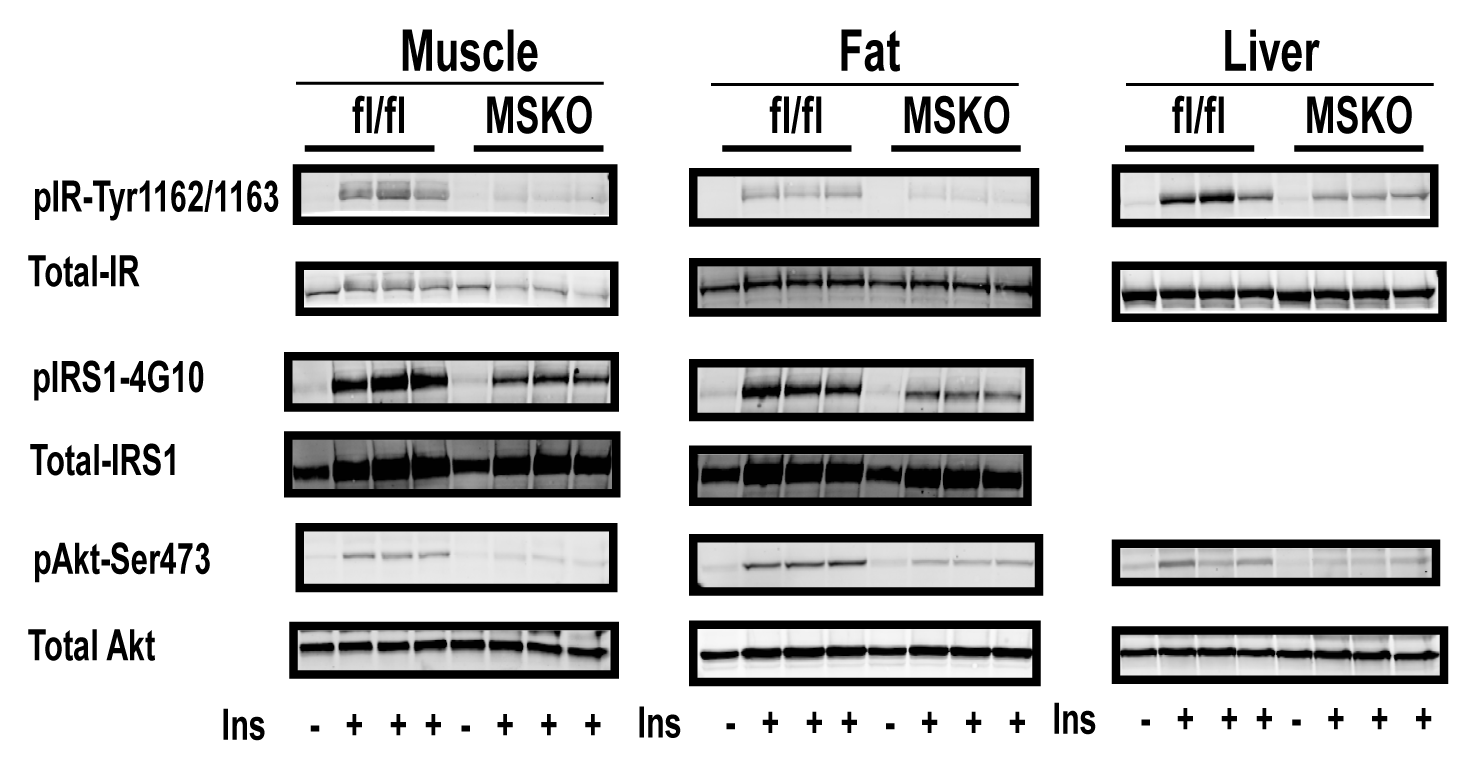

Supplement: Figure S7 — MSKO mice exhibit impaired insulin signaling in muscle, fat, and liver. Insulin signaling study was conducted in male MSKO and fl/fl mice fed HF diets for 24 weeks as described in Materials and Methods. Gastrocnemius muscle, epididymal fat and liver were collected and tyrosyl phosphorylation of IR at Tyr1162/1163 and serine phosphorylation of Akt at Ser-472 were measured by western blotting analysis. Tyrosyl phosphorylation of IRS1 was measured by immunoprecipitation followed by western blotting. (TIF) [file pone.0049935.s007.tif]

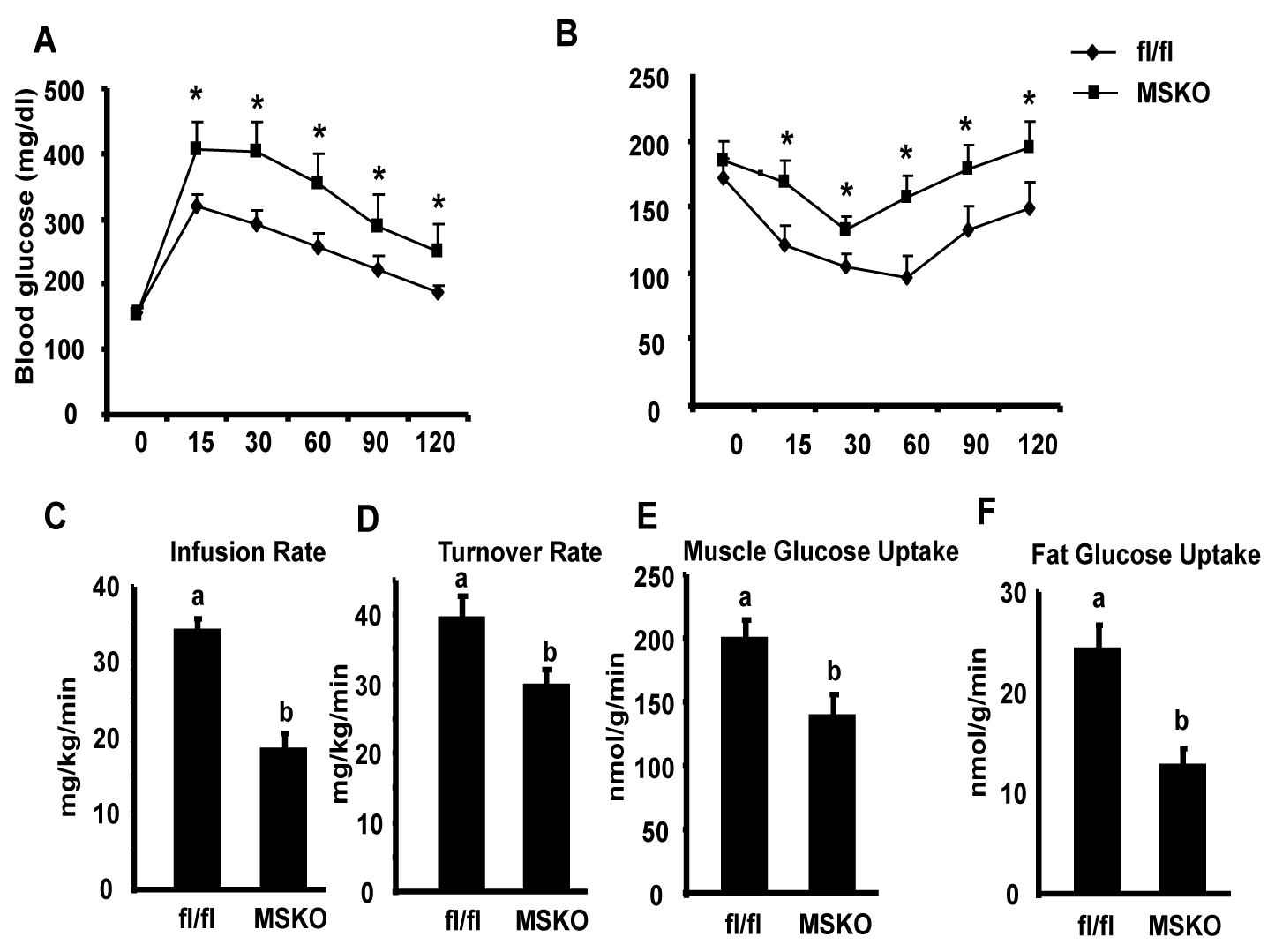

Supplement: Figure S8 — MSKO mice develop insulin resistance on HF diets. (A–B), GTT (A) and ITT (B) were performed on male MSKO and fl/fl control mice fed HF diets for 24 weeks. n = 8–12, *p<0.05 vs. fl/fl. (C–F), Glucose infusion rate (C), glucose turnover rate (D), muscle glucose uptake (E), and fat glucose uptake (F) in male MSKO and control fl/fl mice during hyperinsulinemic-euglycemic clamp study. Hyperinsulinemic-euglycemic clamp was performed as described in Materials and Methods. Data are expressed as mean ± SE, n = 5–6. Groups labeled with different superscripts are statistically different from each other, p<0.05. (TIF) [file pone.0049935.s008.tif]

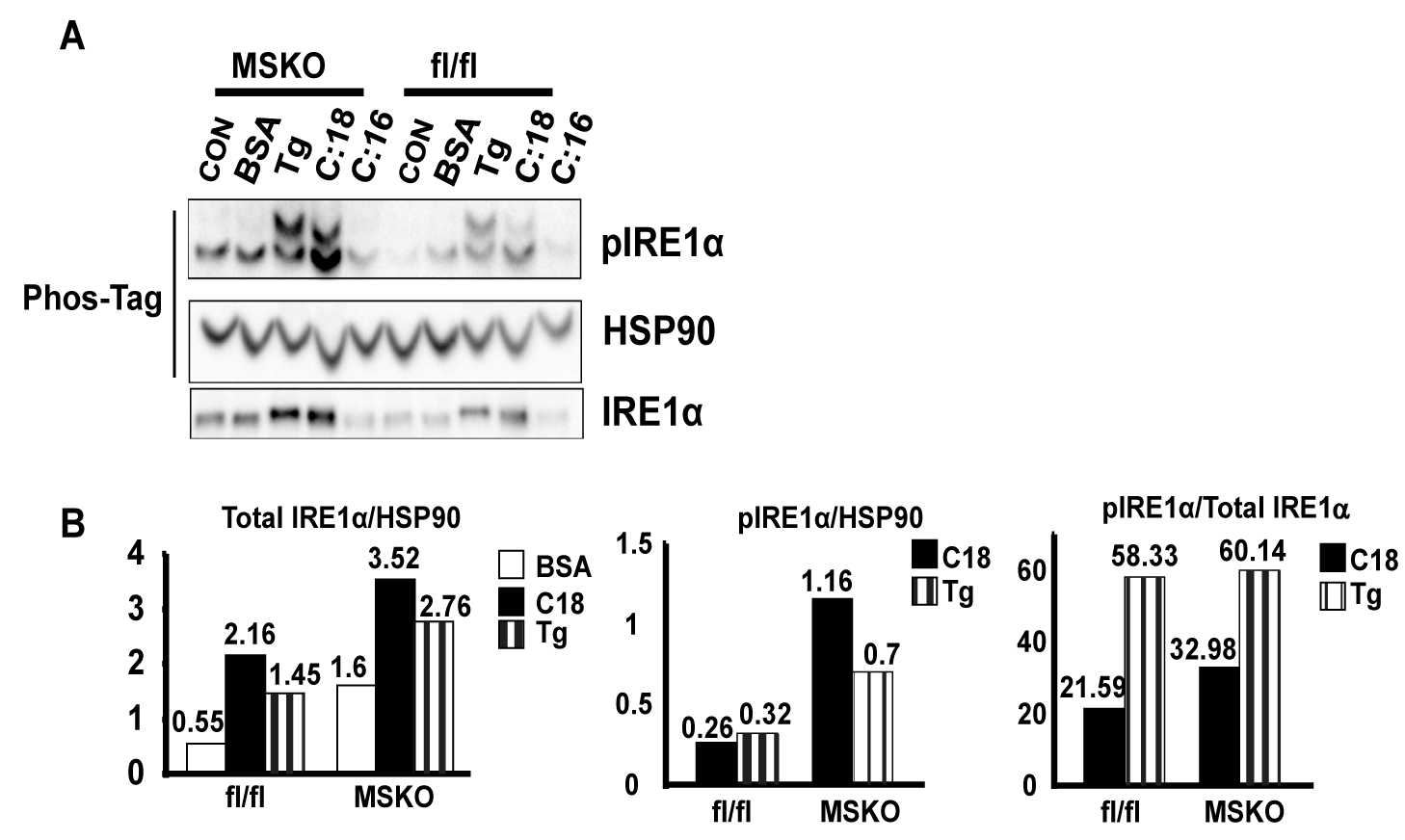

Supplement: Figure S9 — SIRT1 deficiency increases IRE1α protein and phosphorylation levels. (A) Representative blots of IRE1α signaling. (B) Quantitation of IRE1α protein and phosphorylation. Peritoneal macrophages from MSKO or fl/fl mice were isolated by lavage 4 days after intraperitoneal injection of thioglycollate, and were treated with stearate (C: 18, 200 µM) overnight or thapsigargin (Tg) for 2 hours. IRE1α phosphorylation was measured using phos-tag-based approach as described in Materials and Methods. CON: control; BSA: bovine serum albumin. HSP90: heat shock protein 90α (internal control). (TIF) [file pone.0049935.s009.tif]
